# Gender-Specific Neuroimmunoendocrine Response to Treadmill Exercise in 3xTg-AD Mice

**DOI:** 10.4061/2010/128354

**Published:** 2010-10-12

**Authors:** Lydia Giménez-Llort, Yoelvis García, Karla Buccieri, Susana Revilla, Cristina Suñol, Rosa Cristofol, Coral Sanfeliu

**Affiliations:** ^1^Medical Psychology Unit, Department of Psychiatry and Forensic Medicine, Institute of Neuroscience, Autonomous University of Barcelona, Bellaterra, 08193 Barcelona, Spain; ^2^Institute of Biomedical Research of Barcelona (IIBB), CSIC-IDIBAPS, 08036 Barcelona, Spain

## Abstract

The 3xTg-AD mouse develops a progressive Alzheimer's disease- (AD-) like brain pathology that causes cognitive- and neuropsychiatric-like symptoms of dementia. Since its neuroimmunoendocrine axis is likewise impaired, this mouse is also useful for modelling complex age-related neurodegeneration. This study analyzed behavioral, physiological, neurochemical, pathological and immunoendocrine alterations in male and female 3xTg-AD mice and assayed the effects of a short therapy of forced physical exercise at the moderate pathology stage of 6 months of age. Gender effects were observed in most AD-related pathology and dysfunctions. Five weeks of treadmill training produced beneficial effects, such as the reduction of brain oxidative stress and GABA-A receptor dysfunction in males and improvement of sensorimotor function in females. In both sexes, exercise decreased the brain amyloid *β* 42/40 ratio levels. The results highlight the importance of analyzing experimental therapies in both mouse model genders in order to improve our understanding of the disease and develop more appropriate therapies.

## 1. Introduction

Alzheimer's disease (AD) is the leading cause of dementia in people over 65, and it affects more than 20 million people worldwide [1]. No cure has yet been found, and current pharmacological therapy only temporarily ameliorates the symptoms [2]. Recent research has focused on preventive strategies derived from a healthier lifestyle, which may decrease the incidence of AD in the population [3]. One such strategy is physical exercise. Regular physical exercise enhances brain functionality and protects against neurodegeneration through multiple pathways. Regular exercise can attenuate oxidative damage in brain by reducing the ROS production and increasing the antioxidant systems [4]. These effects indicate that exercise could be a preventive tool against neurodegeneration-associated oxidative challenge. Also in brain, exercise can upregulate the expression of growth factors, such as BDNF, VEGF, FGF-2, NGF, and IGF-1 that regulate synaptic plasticity, learning, neurogenesis, and angiogenesis indicating an involvement of exercise in these cerebral processes [5]. On the other hand, exercise is suggested to reduce A*β* accumulation in cortical areas of AD transgenic mouse by increasing proteolytic degradation by proteasome [6]. The exact molecular mechanisms underlying these favorable effects of exercise in brain are not well known, but the MAPK, PI3K, and PI/Akt signaling pathways and the transcription factor CREB have been involved at the molecular level [5]. Exercise also can change the function of glutamatergic systems, increasing both NR2A and NR2B subtypes of the NMDA receptor in the hippocampus [7], which are crucial in learning and memory processes.

The health and psychological benefits elicited by regular physical activity in older adults contribute to healthy aging [8, 9]. Therefore, physical exercise is a potential intervention to preserve or ameliorate cognitive function and behavior in AD. Indeed, exercise is associated with a reduced incidence of AD in the at-risk population [10]. Moreover, improved cognition through exercise has been described in older adults at risk for AD [11]. Physical exercise programs ameliorate mood [12] and symptoms of depression [13, 14] in AD patients. However, the timing and duration of the exercise required to be effective against disease symptoms, as well as the underlying mechanisms, are not known. Anticipated low adherence of AD patients to exercise regimes could be an obstacle for clinical studies, although this can be overcome, at least in part, by experimental studies. Transgenic mouse models of AD are useful and reliable experimental models for testing anti-AD therapies. Several studies have reported some pathology amelioration in AD transgenic mice as a result of physical exercise, either assaying voluntary exercise with a freely available running wheel [15, 16] or forced exercise with a treadmill [16–19]. However, more studies are needed to understand the effects and consequences of physical exercise intervention on the pathological cascade of AD neurodegeneration. Gender differences have not been extensively explored in the literature yet. In parallel, more studies are also needed to find out differential gender responses both in normal and mutant animals.

This study used the mandatory treadmill exercise paradigm in AD triple transgenic mice (3xTg-AD) [20]. These mice develop age-dependent and progressive neuropathology that includes plaque and tangle pathology [21]. Their associated behavioral disturbances include cognitive and noncognitive symptoms (i.e., Behavioral and psychological symptoms of dementia, BPSD) and other neuronal symptoms that mimic AD dementia [22]. In addition, these mice present a gender-related progression of AD changes [23, 24]. Therefore, 3xTg-AD is a valuable model for preclinical intervention studies. As few as 2–5 weeks of moderate intensity, treadmill running has been demonstrated to promote nerve cell regeneration and improve learning and memory in rodents [25–27]. However, some previous studies of treadmill effects in AD transgenic mice were extended to 12 weeks [17, 19] or 16 weeks [18, 28]. This study evaluates the effects of just 5 weeks of treadmill exercise on the brain and peripheral organ function of 3xTg-AD mice at several levels of study, namely the cellular (oxidative stress), neurochemical (GABA function), physiological (body weight curve, glucose homeostasis), immunoendocrine (involution of thymus, plasma levels of glucocorticoids), behavioral (BPSD-like and cognition impairments), and neuropathological (presence of amyloid and tau pathologies). The effects of exercise on physical functions were evaluated by means of sensorimotor tasks, including muscular strength and coordination. Emphasis was placed on the differential AD-like pathology severity present in male and female 3xTg-AD, which underlines the importance of translating the findings of therapeutic studies in this animal model to AD.

## 2. Materials and Methods

### 2.1. Animals

3xTg-AD mice harboring PS1/M146V, APPSwe, and tauP301L transgenes were genetically engineered at the University of California Irvine, as previously described [20]. Six-month-old male and female 3xTg-AD mice from the Spanish colony of homozygous 3xTg-AD mice established in the Medical Psychology Unit, Autonomous University of Barcelona [22, 29] were used in this study. Genotypes were confirmed by PCR analysis of DNA obtained from tail biopsies. Three to five littermates of the same genotype and sex were maintained (Makrolon, 35 × 35 × 25 cm) under standard laboratory conditions (food and water *ad lib*, 22 + 2°C, 12 h light: dark starting at 08:00). 

Thirty-six 3xTg-AD mice were subjected to five weeks of exercise on a treadmill (see below), after which their behavior was assessed on a series of tests used to screen for behavioral abnormalities in mutant mice [22, 30]: physical condition by body weight and sensorimotor tasks (SMT); BPSD-like behaviors, including the corner test (CT), open-field (OF), dark-light box (DLB), and T-maze (TM); several tasks to assess spatial learning and memory in the Morris water maze (MWM). Behavior was evaluated using both direct observation and a video-computerized tracking system (SMART, Panlab S.A.) by two independent observers who were unaware of the animal's genotype. Eighteen nontransgenic (non-Tg) mice with the same genetic background were used as a reference group. Experiments were performed under dim white-light conditions (16–20 lux) from 10:00 to 13:00 and in accordance with Spanish legislation on “Protection of Animals Used for Experimental and Other Scientific Purposes” and the European Communities Council Directive (86/609/EEC) on this subject.

### 2.2. Administration of Exercise on the Treadmill

The animals were divided into two sets according to their gender (males/females) and thereafter randomly assigned to one of two treatment groups (exercise, EXE; no exercise or sedentary, SED). Treatments were administered from Monday to Friday, between 9:00 and 19:00 in a soundproof room under dim light. The experimental design was counterbalanced by gender each day (starting with males in the morning and females in the afternoon, and vice-versa on the next day) and per treatment. The sedentary group (without exercise) was placed on the treadmill running apparatus (Scientific Instruments L18706) for the same amount of time as the animals doing exercise. Animals were constantly monitored, and the training of exercise group was slowly increased over time and by speed, that is, from minimum (15 min/day, 5 cm/s) to moderate exercise (30 min/day, 7 cm/s). The treadmill was carefully cleaned between animals, including a 30-min rest period without animals when starting the set of animals of the opposite gender.

### 2.3. Physical and Behavioral Profile of 3xTg-AD

Two days after the end of the administration of exercise, several physical, physiological, and neuroimmunoendocrine parameters were studied and compared to those of non-Tg mice.

#### 2.3.1. Physical Conditions: Weight and Sensorimotor Functions

The body weight of animals was monitored every day before and after the treatments. The weight of intra-abdominal white adipose tissue (WAT) was measured. The contribution of WAT to total body weight was calculated as the percentage of the weight of WAT versus the total body weight. 

Reflexes (*visual reflex *and* posterior legs extension reflex tests*) were measured three times by holding the animal by his tail and slowly lowering to a black surface. The motor coordination and equilibrium were assessed by the distance covered and the latency to fall off a horizontal* wooden rod* (1.3 cm wide) on two consecutive 20 s trials, respectively. In order to increase the difficulty of the task, the test was repeated on a metal *wire rod* (1 cm diameter). Prehensility and motor coordination were measured as the distance covered on the *wire hang test*, which consisted in allowing the animal to cling from the middle of a horizontal wire (diameter: 2 mm, length: 40 cm, divided into eight 5 cm segments) with its forepaws for two trials of 5 s and a third 60 s trial. Muscle strength was measured as the time until falling off the wire in the 60 s trial. All the apparatus were suspended 40 cm above a padded table.

#### 2.3.2. Corner Test

Neophobia to a new home-cage was assessed by introducing the animal into the center of the standard cage (Makrolon, 35 × 35 × 25 cm) and counting the number of visited corners and rearings during a period of 30 s. Latency of the first rearing was also recorded.

#### 2.3.3. Open-Field Test

Mice were placed in the centre of the apparatus (home-made, wooden, white, 55 × 55 × 25 cm high) and observed for 5 min. Horizontal (crossings of 5 × 5 cm squares) and vertical (rearings) locomotor activities were recorded for each minute of the test. We also recorded the latency of the sequence of the following behavioral events: initial freezing (latency of initial movement), thigmotaxis or discrimination of unprotected/protected areas in the test (latency of leaving the central 5 × 5 cm square and that of entering in the peripheral ring 5 cm to the walls), and self-grooming behavior (latency, number, and duration of groomings). Defecation was also measured.

#### 2.3.4. Dark-Light Box Test

Anxiety-like behavior was also measured in the dark-light box (Panlab, S.L., Barcelona, Spain). The apparatus consisted of two compartments (black, 27 × 18 × 27 cm; white, 27 × 27 × 27 cm; lit with a 20 W white bulb) connected by an opening (7 × 7 cm). The mice were introduced into the black compartment and observed for 5  min. Latency to enter (all four paws) the lit compartment, the time spent in the lit compartment, and the horizontal (crossings of 3 × 3 cm) and vertical (rearings) activities developed once they were recorded. 

#### 2.3.5. Morris Water Maze Tests

Animals were tested for spatial learning and memory in the MWM [31] consisting of four trials of place learning for spatial reference memory followed by one probe trial and one day of cue learning. In the *place-learning task*, mice were trained to locate a platform (7 cm diameter, 1.5 cm below the water surface) in a circular pool (Intex Recreation Corp. CA, USA; 91 cm diameter, 40 cm height, 25°C opaque water) located in a test room with distal visual cues. The acquisition task consisted of four trial sessions per day, with trials spaced 15 min apart. In each trial, the mouse was gently released (facing the wall) from one randomly selected starting point (N, S, E, or W) and allowed to swim until escaped onto the platform (always in the middle of the SE quadrant). Mice that failed to find the platform within 60 s were placed on it for 20 s, the same period as was allowed for the successful animals. In session 4, one and a half hour after the fourth trial of place learning, the platform was removed from the maze and the mice performed a *probe trial test* of 60 s. On the following day, the animals were tested for *the cue learning* of a visual platform consisting of four trials in one single day. The platform was elevated 1 cm above the water level with its new position (NW) indicated by a visible striped flag (5 × 8 cm), and the distal cues were removed. 

During each trial of the learning tasks the escape latency, the distance traveled, and the mean speed were measured by means of the computerized tracking system (SMART, Panlab S.A., Barcelona, Spain). The number of annulus crossings during the probe trial was also measured.

#### 2.3.6. T-Maze Test

The spontaneous exploratory behavior of mice was tested in a T-shaped maze (with arms 25 cm in length). Animals were placed inside the “vertical” arm of the maze with its head facing the end wall. The performance was evaluated by determining with a chronometer the time elapsed until the animal crossed (four paws criteria) the intersection of the three arms [32].

### 2.4. Intraperitoneal Glucose Tolerance Test

Three days after the end of the behavioral assessment, mice were assayed for glucose tolerance with the intraperitoneal glucose tolerance test (IPGTT) [33]. Baseline glucose was measured from tail vein blood in mice fasted overnight. Mice were then loaded by intraperitoneal injection with a solution of 2 g glucose/kg body weight. Blood samples were subsequently collected 15, 30, and 60 min after glucose administration using a standardized glucometer. Results are expressed as mg/dL.

### 2.5. Glucocorticoid Analysis

Mice were sacrificed by decapitation. Samples of about 1 mL of whole trunk blood were collected at the time of sacrifice into heparinized tubes and centrifuged immediately at 10,000 ×g for 2 min. The plasma obtained was stored at –20°C. Corticosterone content (ng/mL) was analyzed using a commercial kit (Corticosterone EIA Immunodiagnostic Systems Ltd, Boldon, UK) and ELISA EMS Reader MF V.2.9-0.

### 2.6. Target Samples

Brain was dissected to obtain hippocampus, cerebral cortex, and cerebellum. Tissue samples were stored at −80°C for further biochemical analysis (see below). The size (weight in mg) and relative size (% versus body weight) of the thymus were recorded as an indirect measure of the putative impairment (involution) of the peripheral immunological system [24].

### 2.7. Immunohistochemistry

The hemibrain of four animals per group was fixed by immersion in 4% paraformaldehyde for 48 h. Paraffin-embedded brain sections were cut at 8 mm. Sections were autoclaved in citrate buffer pH 6 for 10 min to expose the epitopes. The endogenous peroxidase activity was quenched for 15 min in 1% H_2_O_2_. Sections were immunostained with antiamyloid *β* (A*β*), clone 4G8 (Covance, Emeryvlle, CA, USA) or anti-paired helical filament tau (PHF-tau), clone AT180 (Thermo Scientific, Rockford, IL) at dilutions of 1 : 50 and 1 : 100, respectively, both overnight at 4°C. After subsequent washes to remove primary antibody excess, sections were incubated with the appropriate biotin-conjugate secondary antibody for 1 h at room temperature. Sections were developed with diaminobenzidine (DAB) substrate using the avidin-biotin horseradish peroxidase system (Vector Laboratories, Inc.).

### 2.8. Amyloid *β* ELISA

Cerebral cortex was homogenized in a buffer containing 50 mM Tris, 400 mM NaCl, 2 mM EDTA, 0.1% triton X-100, 2% bovine serumalbumin, and a cocktail of protease inhibitors (Complete, Calbiochem). Samples were centrifuged at 12,000 ×g for 7 min at 4°C, and supernatants were collected and used to detect A*β* by ELISA. Both A*β*40 and A*β*42 soluble levels were measured by sandwich ELISA [34]. Antibody clone 6E10 against A*β*1–17 (Chemicon, Temecula, CA, USA) was used as a capture antibody and rabbit polyclonal anti-A*β*40 and anti-A*β*42 (Chemicon) as detection antibodies. After incubation for 3 h, wells were washed, and a horseradish peroxidase-conjugated antirabbit (GE Healthcare, UK**)** was added. Wells were washed with phosphate-buffered saline (PBS), and Quantablue reagent (Pierce, Rockford, IL, USA) was added. Samples were then measured at 320 nm using a plate reader (iEMS Reader MF; Labsystems, Vantaa, Finland).

### 2.9. [^3^H] Flunitrazepam Binding Assay

Synaptic membrane preparations were obtained from mice cerebral cortices that had been stored frozen at −80°C. Brain samples were thawed and homogenized in 50 volumes (w/v) of Tris-HCl buffer (50 mM, pH 7.4) and then centrifuged at 20,000 ×g, at 4°C, 20 min. The pellet was resuspended in 1.5 mL of the same buffer and again centrifuged at 20,000 ×g, at 4°C, 20 min. This procedure was repeated five times. The last suspension was frozen at −80°C for at least one day. For the binding assays, the samples were thawed and centrifuged, and the membranes were then resuspended in 1 mL of 50 mM Tris-HCl buffer/NaCl 200 mM. Proteins were measured in 200 *μ*L following the Bradford method. Membranes were then incubated with 1 nM [^3^H] flunitrazepam for 30 min at 25°C. Binding obtained in the presence of 20 *μ*M diazepam was considered to be nonspecific. After incubation the suspensions were rapidly filtered in a Brandel (Gaithersburg, MD, USA) and washed through Whatman GF/B filters (435 *μ*m). The amount of bound radioactivity was determined following the addition of 2 mL of OptiPhase “Hisafe” scintillation cocktail in a Wallac 1414 Winspectral liquid scintillation counter (Perkin Elmer, Waltham, MA). Radioligand binding data were subjected to Scatchard analysis in order to obtain the neurochemical variables for binding site density (Bmax, in pmol/mg protein) and binding affinity (Kd, in nM).

### 2.10. Lipid Peroxidation, Glutathione Peroxidase, and Superoxide Dismutase Assays

For lipid peroxidation and enzymatic assays, 100 mg of cerebral cortex tissue was sonicated for 30 s in 1 mL of ice-cold 50 mM potassium phosphate buffer containing 1 mM EDTA pH 7.4 and before being centrifuged at 12,000 ×g for 30 min at 4°C. The supernatants were collected and stored at −80°C until assay. Enzymatic activities and lipid peroxidation were determined as described previously [35]. Peroxidation of lipids was measured by determining malondialdehyde and 4-hydroxyalkenal (MDA + 4-HAD) using a Lipid Peroxidation Assay Kit from Calbiochem (EMD Biosciences Inc., Darmstadt, Germany). Glutathione peroxidase (GPx) activity was determined by measuring spectrophotometrically the rate of NADPH oxidation in the presence of hydrogen peroxide. Superoxide dismutase (SOD) activity was measured using the SOD assay kit Ransod (Randox Laboratories Ltd, Crumlin, UK), based on the xanthine*⁄*xanthine oxidase system. After total SOD (Cu/Zn SOD and Mn SOD) was determined, samples were again analyzed in the presence of 500 *μ*M KCN to inhibit CuZn SOD and to obtain Mn SOD activity. SODCuZn activity was obtained by subtracting Mn SOD activity. Proteins were measured in 10 *μ*L of supernatants following the Bradford method.

### 2.11. Reduced Glutathione and Oxidized Glutathione Assays

For reduced glutathione (GSH) and oxidized glutathione disulphide (GSSG) assays, 200 mg of cerebral cortical tissue was sonicated in 1 mL of 3.3% sulphosalicylic acid. Acid homogenates were centrifuged at 12,000 ×g for 30 min at 4°C, and supernatant fractions were collected and stored at −80°C until assay. The levels of GSH and GSSG were determined using an enzymatic assay [36] that is essentially a modification of Tietze's recycling method. Samples for GSSG determination were previously incubated at room temperature with 2 *μ*L of 2-vinyl pyridine per 100 *μ*L sample in order to conjugate any GSH present in the sample, such that only GSSG was recycled to GSH. Both GSH and GSSG samples were then neutralized by the addition of 6 *μ*L of triethanolamine. For total glutathione determination, 50 *μ*L of each sample was mixed with 100 *μ*L of 100 mM sodium phosphate/1 mM EDTA buffer containing 1 mM dithiobisnitrobenzoate, 20 U/mL glutathione reductase, and 1 mM *β*-nicotinamide adenine dinucleotide phosphate (NADPH). The kinetics of the formation of 5-thio-2-nitrobenzoic acid was immediately recorded at 30°C and 405 nm, every 15 s over a 5-min period.

### 2.12. Statistics

Statistical analyses were performed using SPSS 12.0 software. Two-way ANOVA was performed using gender and treatment as the independent variables for detecting the respective effects. Post hoc comparisons were carried out using a post hoc Duncan's test, unless otherwise stated. Temporal courses were analyzed using repeated-measures ANOVA (RMA), and when comparing two time points, paired *t*-test was used.

## 3. Results

### 3.1. Physical and Behavioral Profile of 3xTg-AD

**Figure 1:**
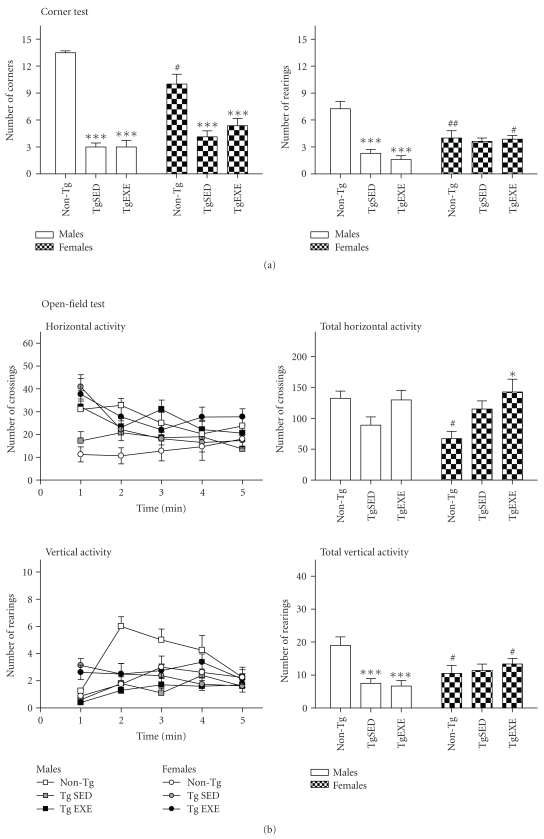

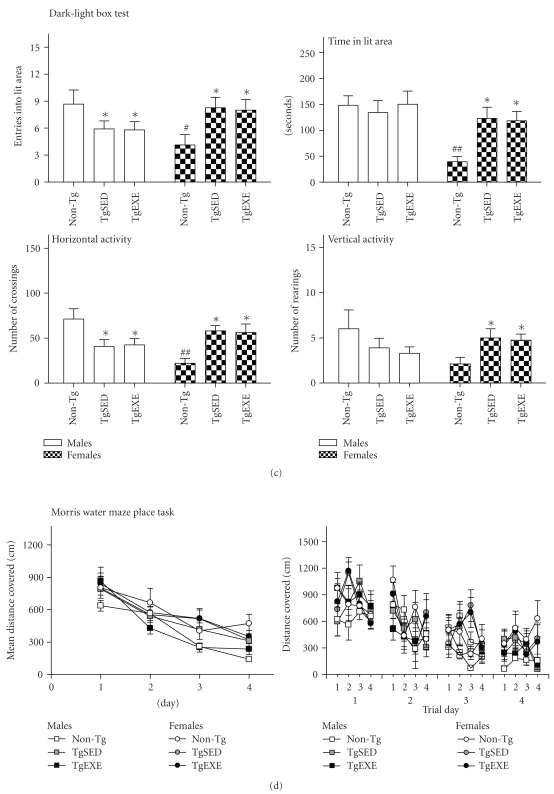
All the behavioral tests do consistently refer to presence of BPSD-like symptoms as a characteristic of early stages of the disease in 3xTg-AD mice, with an important gender component. The effects of exercise are gender dependent. (a) The neophobia in the corner test was increased in both male and female 3xTg-AD mice. (b and c) The anxious profile could be also observed in several variables measured by the open-field test and dark-light box. (d) In the Morris water maze, changes in the swim speed and motivation could be recorded and were mainly important in females (see complementary data in Tables 1(a) and 1(b)). Values are the mean ± SEM, *n* = 8–10. Statistics: *P < .05, **P < .01, and ***P < .001 compared to the corresponding non-Tg;^#^ *P* < .05, ^##^ *P* < .01 compared to the corresponding TgSED.

**Figure 2:**
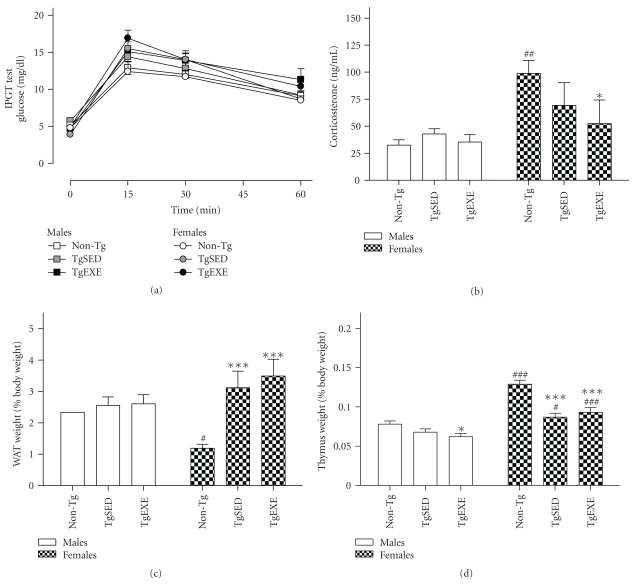
Peripheral physiological-immunoendocrine function is differentially impaired in male and female 3xTg-AD mice and is not ameliorated by treadmill exercise. (a) The IPGT test, indicating plasma glucose levels after an ip injection of glucose, denoted lower tolerance in female 3xTg-AD (see text for statistics). (b–d) Immunoendocrine function as measured by corticosterone plasma levels (b), white adipose tissue (WAT) weight (c), and thymus weight (d) was better in females than in males. Values are the mean ± SEM, *n* = 8–10. Statistics: *P < .05 and ***P < .001 compared to the corresponding non-Tg;^#^ *P* < .05, ^##^ *P* < .01, and ^###^ *P* < .001 compared to the corresponding TgSED.

Tables 1(a) and 1(b), Figures 1 and 2(c) summarize the results obtained in the physical and behavioral assessments of 3xTg-AD mice.

#### 3.1.1. Weight

On a general basis, clear body weight differences could be observed among the different groups with higher weight recorded in males than females in both non-Tg and 3xTg-AD mice, while the transgenic genotype involved a significant increase of weight (see Table 1(a), ANOVA, *F*(5,46) = 29.37, *P* < .001, post hoc Duncan's test, *P* < .05) which was independent of the gender or treatment. The contribution of WAT to total body weight in the non-Tg mice was smaller in females (0.3 ± 0.0 g) than in males (0.8 ± 0.0 g). This gender-dependent effect was completely lost when genotype was considered, as the weight of WAT was exactly the same in both genders of 3xTg-AD mice (M TgSED: 1.0 ± 0.1 g, F TgSED: 1.0 ± 0.0 g, M TgEXE: 1.1 ± 0.2, F TgEXE: 1.0 ± 0.2 g) independent of the treatment. As illustrated in Figure 2(c), the contribution of WAT to total body weight (% versus total weight) was similar in all males independent of genotype or treatment but significantly higher in both groups of females as compared to the non-Tg female group [ANOVA, *F*(5,46) = 5.09, *P* < .001].

When the weight curve was considered, the repeated-measures ANOVA indicated a global effect of the “day” factor (*F*(24,32) = 7.521, *P* < .001) which was dependent on gender (“gender” and “day × gender” interaction effects, *F*(1,32) = 25.163, *P* < .001 and *F*(24,32) = 5.979, *P* < .001, resp.) with a 9.3% statistically significant reduction of weight in females across the test (paired *t*-test, *t* = 5.774, *d* *f*  15, *P* < .001). However, there was no influence of the “exercise” factor on weight (*F*(24,32) = 0.939, *n.s*.).

#### 3.1.2. Sensorimotor Tasks

Two-way ANOVA showed gender-dependent differences on most of the tasks (gender, all *F*'s(1,35) > 4,488, *P* < .05) and a clear beneficial effect of exercise (“exercise,” all *F*'s(1,35) > 4,313) on the distance covered on both rod tests (coordination), with “gender x exercise” interaction effects (“gender × exercise,” all *F*'s(1,35) > 5,088). The post hoc Duncan's test revealed that all females exhibited longer latencies to fall (equilibrium) than did males when the complexity of the task was increased (metal rod test) as well as longer latencies to fall (strength) and more distance covered (coordination and equilibrium) on the 60 s trial of the hanging test. The benefit of exercise had a strong gender component, with the group of treated females showing statistically significant differences with respect to the other three groups in terms of the distance covered on the rod tests (coordination and equilibrium).

#### 3.1.3. Corner Test

As illustrated in Figure 1(a), the normal behavioral pattern exhibited by NTg mice clearly differed between genders, with females being more neophobic than males. On the other hand, two-way ANOVA also showed gender effects in both horizontal (number of corners) and vertical activities (number) (all *F*'s(1,35) > 6.145, *P* < .01). Latency of rearings (see Table 1(a)) was influenced by all factors (genotype, gender, treatment, and gender × treatment interaction effects; all *F*'s(1,35) > 4.846, *P* < .05 and *F*(3,34) = 9.752, *P* < .001, resp.). Exercise did not induce modifications on the total number of these behaviors but modified its onset (as mentioned above) and showed interaction effects with gender in the number and latency of rearings (all *F*'s(3,34) > 6.082, *P* < .01).

#### 3.1.4. Open-Field Test

Figure 1(b) shows the time course and total activity developed in the open-field test, while in Table 1(b), the sequence of behavioral events is indicated. 

In the non-Tg mice males and females mainly differed in the initial horizontal and vertical activities developed in the test, the latency to reach the peripheral area. Overall, that also resulted in a total higher activity in males than females (all *P* < .05). No differences in self-grooming or defecation were recorded.

When the genotype was considered, it was observed that the behavioral sequence of events was faster developed by 3xTg-AD mice, reaching the statistical significance in most of the studied variables in the groups with exercise (see Table 1(b) and Figure 1(b)). Accordingly, the first minute of the test was the most sensitive to show gender (all *F*'s(1,25) > 5.104, *P* < .05) and genotype differences, with TgSED and TgEXE animals being more active than their non-Tg counterparts (males, all *F*'s(2,21) > 5.375, *P* < .01; females, all *F*'s(2,25) > 3.750, *P* < .01). Also, the horizontal and vertical activities developed during the first minute of the test was different between males and females. However, when the total activity developed during the test was considered, a genotype × gender interaction effect (all *F*'s(3,34) > 3.125, *P* < .05) was found. Thus, genotype differences were maintained in the female groups (TgSED and TgEXE) but were inversed (vertical activity) in the male groups (TgSED and TgEXE). Exercise increased self-grooming behavior was observed in male TgEXE.

#### 3.1.5. Dark-Light Box Test

As in the previous tests (see Table 1(b) and Figure 1(c)) gender-dependent differences between non-Tg males and females were observed and then disappeared in the 3xTg-AD genotype. TgSED and TgEXE males exhibited reduced number of entries into the lit area, and once there they developed less locomotor activity and increased the number of defecations (all *F*'s(2,25) > 3.750, *P* < .05). Oppositely, female TgSED and TgEXE increased their presence in the lit area as it was consistently shown in all the variables measured in the test (all *F*'s(2,21) > 3.508, *P* < .05). 

#### 3.1.6. Morris Water Maze Tests

Figure 1(d) illustrates the “day-by-day” and “trial-by-trial” acquisition curves. Results are expressed in distances because we found gender, genotype, and gender × genotype differences in the swim speed (see Table 1(b)). In the non-Tg mice, mean speed of females was slower than males, oppositely of what was found in 3xTg-AD mice. Besides, mean speed of 3xTg-AD was higher than that of non-Tg counterparts (*F*(5,46) = 3.18, *P* < .05), mainly in females (see Table 1(b)). 

All the animals exhibited a similar acquisition curve (day effect, *F*(3,152) = 31.0, *P* < .001), but in the males the reduction of the mean distance covered to find the platform along the four days of the test was faster than in females (*F*(5,152) = 2.483, *P* < .05). Still, no differences could be found in the annulus crossing during the probe trial, except for a general gender effect (*F*(3,35) = 31, *P* < .001).

In the cue learning task gender differences in the non-Tg groups were also found in the mean distance covered and the speed, with non-Tg males taking longer and being faster than non-Tg females (see Table 1(b), *t*-test, 14 *df*, *P* < .05 in both cases). This gender effect was not observed among the 3xTg-AD groups.

#### 3.1.7. T-Maze

Differences in the latency to reach the intersection of the T-maze were found (*F*(5,46) = 2.31, *P* < .05) with the highest values spent by non-Tg females as compared with their male non-Tg and female 3xTg-AD counterparts. No differences were due to exercise.

### 3.2. Glucose Tolerance

Results of the IPGTT test are shown in Figure 2(a). Female 3xTg-AD mice showed decreased glucose tolerance as related to non-Tg mice. ANOVA showed an effect for female glucose curves (*F*(2,84) = 6.764, *P* < .005) with the peak of glucose levels (15 min of the IPGTT test) in female 3xTg-AD mice, both sedentary and exercised groups, being higher than those measured in non-Tg female mice (*P* < .05). On parallel both groups of male 3xTg-AD mice showed a trend to impaired glucose tolerance albeit it did not reach statistical significance.

### 3.3. Immunoendocrine Status

Corticosterone plasma levels are shown in Figure 2(b). Weight of WAT and thymus are shown in Figures 2(c) and 2(d). Other weighed organs did not show any significant differences between the experimental groups. There was a “gender” effect on corticosterone levels (*F*(1,52) = 1.18, *P* < .005) and thymus weight (*F*(1,46) = 78.02, *P* < .001). There was a “genotype” effect on thymus weight (*F*(2,46) = 20.68, *P* < .001) and WAT weight (*F*(2,46) = 8.051, *P* < .001), with “genotype × gender” interaction effects in both weights (*F*(2,46) = 5.996, *P* < .005 and *F*(2,46) = 5.042, *P* < .01, resp.). non-Tg females showed higher corticosterone and thymus weight and lower fat than did males. 3xTg-AD females showed a decrease in corticosterone and thymus weight and an increase in fat as compared to non-Tg females. However, 3xTg-AD females showed a better immunoendocrine status than did the corresponding males, as shown by thymus weight. Treadmill exercise had a minimal effect on these parameters.

### 3.4. Brain Amyloid Pathology

**Figure 3:**
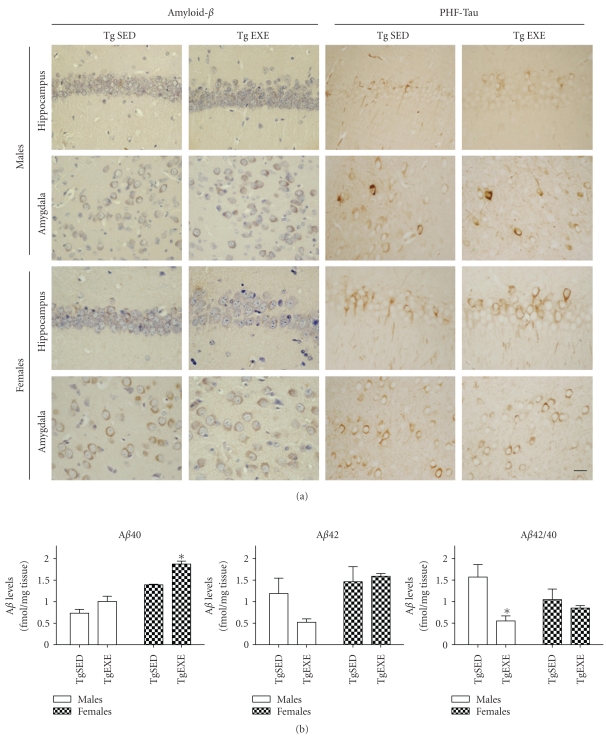
Brain pathology in male and female 3xTg-AD sedentary mice (TgEXE) is partially protected against by treadmill running exercise (TgSED). (a) Representative microphotographs of hippocampus CA1 and basolateral amygdala showing amyloid-*β* and PHF-tau immunoreactivity using antibodies 4G8 and AT180, respectively. 4G8 reacts against amino acids 1–17 of amyloid *β*. AT180 detects phosphorylated tau at the threonine 321 residue. No changes in intraneuronal amyloid *β* or PHF-tau were induced by exercise. Scale bar = 20 *μ*m. (b) Levels of soluble amyloid *β* 40 (A*β*40) and amyloid-*β* 42 (A*β*42) in cerebral cortex as determined by sandwich ELISA. Treadmill exercise induced a protective change by decreasing the A*β*42/A*β*40 ratio (A*β*42/40) (ANOVA, *P* = .0110). Values are the mean ± SEM, *n* = 3-4. Statistics: *P < .05.

In order to assess AD pathology, we performed A*β* and PHF-tau immunostaining in sections of male and female 3xTg-AD mice that had been maintained under sedentary or forced exercise conditions (Figure 3(a)). Immunoreactivity of 4G8 and AT180 was evident in hippocampus and amygdala neurons. Treadmill exercise did not decrease 4G8 and AT180 immunoreactivity in any of the 3xTg-AD sex groups. Further, we measured soluble A*β*40 and A*β*42 levels in cerebral cortex of male and female 3xTg-AD mice (with and without exercise) by using sandwich ELISA (Figure 3(b)). Both peptides showed higher levels in females than in males. Two-way ANOVA analyses showed a significant effect of “gender” on A*β*40 (*F*(1,9) = 64.97, *P* < .005) and A*β*42 (*F*(1,9) = 8.159, *P* = .05). Exercise induced an increase in A*β*40 level and a decrease in the A*β*42/A*β*40 relationship in female mice. Two-way ANOVA showed a significant effect of “exercise” on the A*β*42/A*β*40 ratio (*F*(1,19) = 10.17, *P* < .05), but no “gender” effect. We therefore analyzed all animals together using a two-tailed Student's *t*-test, which confirmed that treadmill exercise decreased the A*β*42/A*β*40 ratio in 3xTg-AD (last graph in Figure 3(b)).

### 3.5. GABA Function Alteration

The effects of treadmill exercise on GABA-A receptor functionality were assayed by measuring [^3^H] flunitrazepam binding parameters in male and female non-Tg mice, 3xTg-AD without exercise and 3xTg-AD with treadmill exercise (Table 2). We observed that the density of the [^3^H] flunitrazepam binding sites (Bmax) was unchanged in both male and female 3xTg-AD compared to non-Tg. In relation to the dissociation constant (Kd), there were no binding affinity changes among any of the three groups of female mice. In contrast, 3xTg-AD males that had not practiced exercise showed a lower Kd of flunitrazepam than did non-Tg mice (Kd increased 2.15-fold in 3xTg-AD versus non-Tg), whereas those subjected to one month of forced exercise had recovered control affinity levels.

### 3.6. Oxidative Stress

**Figure 4:**
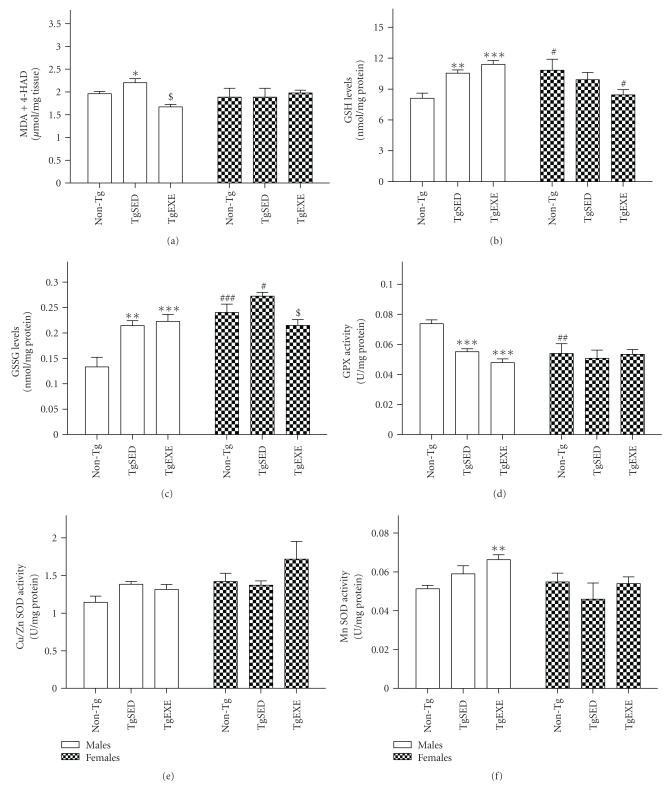
Oxidative stress in the cerebral cortex of sedentary male 3xTg-AD mice (TgSED) is partially ameliorated by treadmill running exercise (TgEXE), whereas 3xTg-AD females show less alteration. Tissue of sedentary nontransgenic mice (non-Tg) was analyzed as a reference. (a) Lipid peroxidation levels expressed as malondialdehyde (MDA) plus 4-hydroxyalkenal (4-HAD). (b) Levels of reduced glutathione (GSH). (c) Levels of oxidized glutathione (GSSG). (d) Glutathione peroxidase (GPx) enzymatic activity. (e) SOD-CuZn enzymatic activity. (f) SOD-Mn activity. Data represent the mean ± SEM of 4–8 animals, tested in duplicate. Statistics: *P < .05, **P < .01, and ***P < .001 compared to non-Tg; ^#^ *P* < .05 compared to TgSED.

The effects of exercise on the disturbed redox homeostasis of 3xTg-AD were studied by analyzing brain cortex lipid peroxidation levels and the status of the main antioxidant cell systems. Lipid peroxidation levels (Figure 4(a)) were increased in 3xTg-AD males as compared to non-Tg, but this was not the case in females. Exercise significantly reduced male lipid peroxidation. GSH and GSSG levels (Figures 4(b) and 4(c)) were significantly increased in 3xTg-AD males as compared to non-Tg, and these alterations were not changed by treadmill exercise. non-Tg females had higher glutathione levels than did males. Levels were also high in 3xTg-AD females, but significantly reduced by exercise. GPx activity (Figure 4(d)) was significantly decreased in 3xTg-AD males as compared to non-Tg, and this was not changed by exercise. non-Tg females had lower GPx levels than did males. SOD-CuZn activity (Figure 4(e)) was not changed among the several groups of male and female mice, but there was increased SOD-Mn activity (Figure 4(f)) in 3xTg-AD exercised males compared to non-Tg.

A two-way ANOVA for 3xTg-AD animals indicated an effect of “gender” in SOD-Mn (*F*(1,16) = 13.13, *P* < .005), GSH (*F*(1,16) = 90.70, *P* < .001), GSSG (*F*(1,18) = 46.80, *P* < .001), and GPx (*F*(1,18) = 23.93, *P* < .001) as well a “gender × exercise” interaction with respect to GSH (*F*(1,16) = 5.479, *P* < .05). Accordingly, there was a difference between 3xTg-AD sedentary males and females in SOD-Mn (*P* < .05), GSH (*P* < .001), GSSG (*P* < .001), and GPx (*P* < .05), but in exercised male and female 3xTg-AD the difference was only observed for GSH (*P* < .001), GSSG (*P* < .001), and GPx (*P* < .001). In contrast, there were no differences between nontransgenic males and females in any of the redox parameters analyzed. The correlation analysis showed a direct relationship between SOD-Mn activity and both GSH and GSSG levels as well as a negative relationship between SOD-Mn activity and lipid peroxidation levels and GPx activity.

## 4. Discussion

In this work we have performed a wide screening in both genders of 3xTg-AD mice at early stages of the disease and non-Tg mice. Several levels of study have been considered from cellular to behavioral ones, including new areas of interest such as physical condition, glucose homeostasis, immunoendocrine function, and oxidative stress. The main interest of the present results has been the description of differences of gender, specific for both genotypes, as well as the beneficial effects that even a short training with forced exercise can have. Also, the results indicate that interaction effects between gender, genotype, and treatment should be always taken into consideration when assessing the outcome of preventive and/or therapeutic interventions. 

The first studies focused on the physical condition. Expected gender differences on weight between males and females were found in both genotypes. The results do also confirm that the overweight reported from early stages of the disease in male 3xTg-AD mice of the Spanish colony [22] is observed in both genders and that females seem to be more sensitive to some etiological-causal and related factors. Thus, in this strain background (C57BL/6 x 129S non-Tg mice) WAT had a statistically significant smaller contribution to the total body weight in females as compared to males. However, in the 3xTg-AD mice the total amount of WAT was equal in both genders, and both the total amount and the relative contribution of WAT exhibited a three-fold increase with the triple-transgenic genotype, an effect restricted to females. Since overweight and glucose metabolism are closely related, we also studied both basal glycemia and glucose tolerance. Although the overweight animals did not show altered basal glucose levels, the increased peak of glucose observed in both genders of 3xTg-AD confirmed our recent findings suggesting that glucose homeostasis is compromised in 3xTgAD mice from early stages of the disease [37]. The results also suggest that females may be more sensitive than males (statistical significance was only reached in females). In addition, other metabolism derangements can be involved in the increased relative WAT levels in females and the generally increased body weight in 3xTg-AD mice. For instance, leptin-related pathways were reported to be impaired with mice models of AD and leptin treatment ameliorated cognitive loss [38]. The weight of females was also more sensitive to repeated handling or exposure to the apparatus itself, independent of the administration of the treatment, as a decline in the curve of weight was only observed in this gender but not in male mice. We cannot assert to which extent the loss of weight can be related to emotional reactivity as, on general, blood corticosterone levels did not differ between both groups of 3xTg-AD females, and, in any case, the corticosterone levels were lower than that recorded on non-Tg females. Overall, the results indicate a poorer ability to maintain weight and glucose homeostasis in female 3xTg-AD mice already at early stages of the disease. The lack of effects of exercise on weight or glucose tolerance may be explained by the fact that the profile exhibited by animals submitted to exercise could be classified as that of “low runners” and therefore is not enough for such expected beneficial effects. In fact, the physical condition of both male and female 3xTg-AD was observed to be lower than that expected for animals of its age as other strains of mice can be successfully trained in three different treadmill exercise conditions from minimum (15 min/day, 5 cm·s^−1^, 0° slope) to moderate exercise (60 min/day, 10 cm·s^−1^, 0° slope) to exhaustive exercise (60 min/day, 25 cm·s^−1^, 20° slope). Here, no slope, low initial speed, and a slow increase of speed and duration of exercise had to be used to avoid animals to get physically exhausted. Moreover, it was observed that animals only run when approaching the electric foot-shock panel, but once reaching the opposite side of the apparatus they stopped. The pause persisted until arriving to the proximity of the panel when they felt encouraged again to run to avoid the negative reinforce. Thus, the 3xTg-AD mice could be classified as “low or nonrunners” according to the “pause number” criterion [38].

The behavioral profile which was aimed to assess not only cognitive but also BPSD-like behaviors which we have described can be already observed from early stages of the disease in 3xTg-AD male mice [22, 29]. All the results obtained in the different behavioral tests (corner, OF, and DLB) consistently indicated gender-dependent differences in anxiety-like behaviors in non-Tg mice; that is, non-Tg females showed increased anxiety as measured by reduced number of horizontal and vertical activities in the three tests, increased latencies of some behavioral events in the open field, and reduced entrance and time spent in the lit area of the dark-light box as compared to non-Tg males.

The results also showed that the transgenic behavioral phenotype was characterized by increased BPSD-like behaviors. Thus, both male and female 3xTg-AD mice exhibited a strong increase of neophobia in the corner test which was nicely observed in the horizontal component (corners), while the vertical activity levels of 3xTg-AD mice did not differ from that shown by non-Tg females. The results in the open field do confirm the increase of anxiety-like behaviors which imply a faster sequence of events related to thigmotaxis or preference for protected areas in the test (leaving the central square to enter in the peripheral ring) and increased horizontal activity (hyperactivity in females). In contrast, the vertical activity in the three groups of both males and females found a strong parallelism with that exhibited in the corner tests and that to be observed in the dark-light box on the next day. In the DLB, the BPSD-like behaviors exhibited by both groups of 3xTg-AD mice were gender dependent. Disinhibitory behavior was observed in TgSED and TgEXE females as compared to non-Tg females. The BPSD-like behavior in males was manifested as increased anxiety (reduction in the number of entries and therefore of horizontal and vertical activities, increased defecatory behavior) although in previous works we have also been able to observe the disinhibitory effect in males [22]. 

When the animals were tested for spatial learning and memory in the different paradigms of MWM, no differences could be found on their learning and memory abilities. However, the main finding was an increased speed during all the days of the tests in the female groups (TgSED and TgEXE), although in current investigations we have found this effect also in 3xTg-AD males. The speed was reduced in the cue learning task albeit only reached statistical significance in non-Tg mice and 3xTgAD females (TgSED and TgEXE), but it is indicative of genotype and genotype-dependent differences in motivation or emotionality, in agreement with the increase of BPSD-like behaviors observed in the other tests. Genotype-dependent differences in thermoregulation [22] could also account for the observed differences in swimming speed. These results are also aware about the relevance of analysing the trajectories on parallel to the traditional latency measurement.

In a previous study [24] we have described gender-dependent neuroimmunoendocrine disfunction in 3xTg-AD at advanced stages of the disease (15 month of age). In this work, the weight of thymus was used as an indirect indicator of immunological functional state, with involution of thymus showing a correlation with differences in immunological function [39] and immunological aging [40]. As expected, the total weight of thymus was higher in females than in males, and this gender difference was more obvious when its relative weight was considered, suggesting a better immune function in the female gender. Interestingly, although this gender-dependent protection was still persistent in 3xTg-AD females, it was strongly reduced as compared to the one exhibited by non-Tg females. Also in males, the transgenic phenotype resulted in involution of the weight of thymus albeit it only reached statistical significance in one of the transgenic groups. Therefore, the present results show an involution of thymus in adult 3xTg-AD mice and suggest that the gender-dependent neuroimmunoendocrine disfunction described in old 3xTg-AD mice [24] may find a clear correlation with impaired immunological function at early stages of the disease, a subject which is under current investigations. Related to this, female 3xTg-AD mice were very fast to reach the intersection of the T-maze as compared to the non-Tg females. This fast performance could be more fight counterpart to the flight copying with stress strategy (slow performance) than has been related to premature accelerated neuroimmunoendocrine system in mice [32].

When considering neuropathological aspects, it is known that at six month of age, 3xTg-AD mice develop intraneuronal A*β* as a characteristic pathological feature of this AD mouse model [20, 21]. A*β* deposits appeared slightly larger in females than in males, in agreement with previous observations in this mouse [41]. These mice showed intraneuronal PHF-tau. At this early age the phosphorylation at Thr321 in intraneuronal tau can be added to that previously described for Thr212, Ser214, and Ser262 in soluble tau [42]. The exercise paradigm used (forced treadmill running) did not ameliorate A*β* deposits or PHF-tau pathology. Other authors also reported no plaque reduction with a similarly short exercise treatment in a freely available wheel [43, 44]. Brain levels of soluble A*β*40 and A*β*42 at 7 months of age were low in comparison to levels of more than 100 fmol/mg reported at 13 months [20]. Interestingly, the two-way ANOVA showed that A*β* levels were, overall, higher in females than in males. Therefore, this increased level may cause the greater behavioral alterations observed in young adult females compared with males (this study, [41]).

In the neurochemical approach, we considered underlying mechanisms of BSPD-like symptoms that model noncognitive AD deterioration which are yet needed to be further explored in these mice. The GABAergic system is considered to be involved in depressive symptoms in AD [45], and the lowered affinity for flunitrazepam binding found in 3xTg-AD males paralleled that reported in the temporal cortex of AD patients [45]. The GABAergic system is less sensitive to the neurodegeneration process than are other neurotransmitter receptors, and GABA-A receptors are relatively well preserved in AD. However, particular receptor subunits may be altered, leading to compensatory changes or physiological disturbances [46, 47]. Forced exercise reversed the flunitrazepam binding affinity to non-Tg values, although it barely ameliorated the anxiety and depression-like behaviors, as discussed above. Therefore, there is gender dualism in the mechanisms underlying noncognitive behaviors in these AD mice, and BPSD are probably derived from disturbances in several neurotransmitter systems and the subsequent imbalance that is caused [45, 48].

Finally, although conclusive evidence is still lacking, the oxidative stress hypothesis of AD may help to explain the triggering of AD neurodegeneration by environmental or genetic factors [49]. Indeed, AD brain shows an elevation of markers for oxidative damage [50–53], and the prevalence of these markers is higher in initial than later stages of the disease, suggesting an involvement of oxidative stress in AD etiology [54, 55]. Therefore, treatments that help to restore redox homeostasis may delay or ameliorate AD pathology. The presence of brain oxidative stress in 3xTg-AD mice at an early age of 3–5 months (females) has been previously reported [56]. In this study, older mice aged 7 months showed fewer changes in oxidative stress. The main effect found was an increased lipid peroxidation and a derangement of the glutathione system in males. The latter disturbance is consistent with the glutathione metabolism gender alteration in AD male patients [57]. One of the mechanisms involved in physical exercise is the regulation of redox homeostasis. Chronic nonstrenuous exercise decreases oxidative damage and increases antioxidant defenses in brain [4]. Accordingly, 3xTg-AD male rats submitted to treadmill exercise showed a decreased level of lipid peroxidation. Exercise also increased the activity level of SOD-Mn, the first line of defense against mitochondrial oxidative damage. No amelioration of the glutathione system changes was obtained in males, but a decreased oxidization of glutathione was observed in females.

## 5. Conclusions

Physical exercise is a promising intervention against AD, and the use of a good model such as the 3xTg-AD mice will enable the best exercise strategies to be defined. Five weeks of forced treadmill exercise partially protected 3xTg-AD mice against AD-like pathology. However, the results differed according to gender, this being due to the differential pathological phenotype of 3xTg-AD males and females, in agreement with previous reports [19, 20, 58]. Specifically, we found that males showed more homeostasis redox derangement than did females, while females showed greater brain AD pathology compared with males. Peripheral immunoendocrine status was better in females than in males, even though 3xTg-AD females were impaired with respect to non-Tg females. Therefore, the 3xTg-AD mouse models human gender differences in the progression and expression of the disease. The results of this study corroborate the beneficial effects of a short burst of physical exercise at a moderate stage of AD neurodegeneration (7 months of age), even though these effects were rather modest. Studies with voluntary exercise are ongoing [41], as this might induce greater neuroprotective effects than does forced exercise [59]. We speculate that over a short training period the stress associated with the mandatory treadmill exercise may partially interfere with the beneficial aerobic effects.

## Figures and Tables

**Table:** (a) The treadmill exercise exerted beneficial effects on the sensorimotor function which were more relevant in females

	M non-Tg	M TgSED	M TgEXE	F non-Tg	F TgSED	F TgEXE
	(*n* = 8)	(*n* = 10)	(*n* = 10)	(*n* = 8)	(*n* = 8)	(*n* = 8)
Body weight						

*Weight curve*						
Start of week 1 (g)	34.4 ± 0.7	36.0 ± 0.8	39.2 ± 1.8	24.7 ± 1.2	32.3 ± 1.2	32.0 ± 1.4
End of week 5 (g)	34.4 ± 0.4	37.0 ± 0.7	38.4 ± 1.6*	23.8 ± 0.3*	28.8 ± 1.0^#^	29.7 ± 1.2^#^
WAT (% versus body weight)	See Figure 1					

Sensorimotor function						

*Reflex tests*						
Incidence of both reflexes	8/8	10/10	10/10	8/8	8/8	8/8
						
*Wooden rod test*						
Equilibrium (mean latency to fall, s)	8.3 ± 2.2	17.5 ± 1.4	19.2 ± 0.5	14.5 ± 2.1	20.0 ± 0.0	20.0 ± 0.0
Coordination (mean distance, cm)	2.5 + 1.5	3.2 ± 2.0	5.7 ± 2.5	1.4 ± 0.7^#^	5.3 ± 4.3^#^	39.7 ± 8.4^#^
						
*Wire rod test *						
Equilibrium (mean latency to fall, s)	3.0 ± 0.5	9.4 ± 1.9	9.8 ± 1.7	3.5 ± 0.8^#^	15.4 ± 1.6^#^	16.7 ± 1.6^#^
Coordination (mean distance, cm)	0 ± 0	0 ± 0	5.3 ± 4.0	0 ± 0^#^	0.9 ± 0.9^#^	19.7 ± 6.6^#^
						
*Wire hang test (2 trials 5 s)*						
Strength (mean latency to fall, s)	0.9 ± 0.1	4.1 ± 0.4*	4.2 ± 0.3*	0.9 ± 0.3	4.5 ± 0.2*	5.0 ± 0.0*
Coordination (mean distance, cm)	0 ± 0	0.5 ± 0.3	0.6 ± 0.4	0 ± 0	0.3 ± 0.3	1.1 ± 0.3
Elements of support (*n*)	1.1 ± 0	1.5 ± 0.4	1.2 ± 0.3	0.8 ± 0.1	1.25 ± 0.4	2.3 ± 0.4
						
*Wire hang test (1 trial 60 s)*						
Strength (mean latency to fall, s)	4.0 ± 2.6	34.1 ± 7.6*	33.4 ± 6.4*	1.0 ± 0.1	55.7 ± 4.2^#,∗^	55.0 ± 5.0^#,∗^
Coordination (mean distance, cm)	0.3 ± 0.3	2.4 ± 0.6*	3.1 ± 0.5*	0 ± 0	5.0 ± 1.7*	6.7 ± 1.1^#,∗^
Elements of support (*n*)	1.7 ± 0.3	2.4 ± 0.3	2.4 ± 0.3	1.1 ± 0	2.8 ± 0.2	3.0 ± 0.0^#,∗^

Note: non-Tg, nontransgenic mice; TgSED, 3xTgAD mice not exercised; TgEXE, 3xTg-AD mice submitted to a daily treadmill running for five weeks. The body weight, fat composition, and sensorimotor function of 3xTg-AD mice differed from that of non-Tg mice. The treadmill exercise exerted beneficial effects on the sensorimotor function which were more relevant in females. Results are the mean ± SEM, *n* = 8–10 as indicated. Statistics: *P < .05 compared to non-Tg;^#^ *P* < .01 compared to TgSED.

**Table:** (b) BPSD-like behaviors are characteristic of 3xTg-AD mice at early stages of the disease and show an important gender component

	M non-Tg	M TgSED	M TgEXE	F non-Tg	F TgSED	F TgEXE
	(*n* = 8)	(*n* = 10)	(*n* = 10)	(*n* = 8)	(*n* = 8)	(*n* = 8)

Corner test						

Vertical activity (latency, s)	5.0 ± 1.5	16.3 ± 7.4*	19.6 ± 7.9*	8.9 ± 4.7	5.3 ± 1.7	10.5 ± 4.1

Open-field test						

Initial movement (latency, s)	7.2 ± 1.3	3.3 ± 1	1.9 ± 0.6*	13.6 ± 6.7	4.2 ± 1.7	3.0 ± 0.9
Exit of the center (latency, s)	14.2 ± 2.3	10.7 ± 2	5.2 ± 1.1*	30.4 ± 13.7	7.6 ± 2.3	4.4 ± 1.0*
Entrance to periphery (latency, s)	17.0 ± 5.3	21.7 ± 8	10.7 ± 2.5	57.6 ± 16.9^#^	10.6 ± 3.2*	7.8 ± 2.8*
Vertical activity (latency, s)	49.2 ± 6.9	75.0 ± 14	119.7 ± 31.2*	87.5 ± 17.7	29.5 ± 5.3^#∗^	34.2 ± 6.4^#∗^
Self-grooming (latency, s)	173.0 ± 57.0	172.3 ± 18	172.1 ± 18.3	127.8 ± 11.6	136.9 ± 23.5	183.0 ± 21.0
Total self-grooming duration (s)	3.2 ± 1.6	3.9 ± 1.0	6.7 ± 1.3*	4.4 ± 0.9	5.6 ± 1.0	3.0 ± 0.9
Defecation boli (*n*)	2.0 ± 0.3	2.7 ± 0.7	2.8 ± 0.4	3.2 ± 0.5	4.4 ± 0.5	2.9 ± 0.6

Dark-light box test						

Latency to enter into the lit area (s)	16.2 ± 2.4	25.5 ± 5.9	47.4 ± 28.2	39.4 ± 12.3	11.5 ± 2.3*	16.1 ± 5.9
Defecation boli (*n*)	0.2 ± 0.2	4.3 ± 0.7*	4.2 ± 0.5*	2.4 ± 0.4	4.0 ± 1.3	4.4 ± 0.7

Morris water maze tasks						

*Place learning task*						
Mean speed on day 1 (cm/s)	18.9 ± 0.8	21.6 ± 0.9	20.9 ± 0.7	19.3 ± 0.8	22.7 ± 0.7	22.7 ± 1.2
Mean speed on day 2 (cm/s)	20.6 ± 0.6	23.4 ± 1.2	22.7 ± 0.7	19.4 ± 1.1	23.8 ± 0.7	24.0 ± 1.3
Mean speed on day 3 (cm/s)	22.0 ± 0.5	21.4 ± 1.6	23.7 ± 0.7	19.2 ± 1.5	23.3 ± 0.3	23.3 ± 1.4
Mean speed on day 4 (cm/s)	21.7 ± 1.2	20.9 ± 0.8	19.9 ± 1.1	18.0 ± 2.0	23.3 ± 0.7	23.4 ± 1.3
Mean speed (cm/s)	20.8 ± 0.7	21.8 ± 1.0	21.8 ± 0.6	19.0 ± 1.1^#^	23.2 ± 0.4*	23.4 ± 1.2*
*Probe trial*						
Annulus crossings (*n*)	8.0 ± 1.41	8.4 ± 1.5	10.1 ± 1.0	5.6 ± 0.8	6.3 ± 1.1	6.9 ± 0.7
*Cue learning task*						
Mean distance covered (cm)	567.0 ± 62.5	138.2 ± 42.4	399.7 ± 149.9	235.9 ± 71.0^#^	312.8 ± 162.6	293.7 ± 96.6
Mean speed (cm/s)	19.1 ± 0.5	19.3 ± 1.3	20.4 ± 0.7	14.8±1.6^#p^	18.8 ± 1.1^p^	19.0 ± 1.4^p^

T-maze						

Performance (latency, s)	8.7 ± 1.2	12.9 ± 1.7	11.3 ± 1.3	20.0 ± 5.9^#^	9.9 ± 1.2*	7.8 ± 1.0*

Note: non-Tg, nontransgenic mice; TgSED, 3xTgAD mice not exercised; TgEXE, 3xTg-AD mice submitted to a daily treadmill running for five weeks. The 3xTg-AD mice exhibited several behavioral changes indicative of increased neofobia, emotionality, anxiety-like behavior, hyperactivity, and impulsivity, which were mainly observed in females. The exercise did not induce major changes but showed gender-dependent effects. Results are the mean ± SEM, *n* = 8–10 as indicated. Statistics: *P < .05 compared to non-Tg;^#^ *P* < .01 compared to TgSED, ^p^compared to mean speed on place learning task *P* < .05.

**Table 2:** Treadmill exercise recovers the [^3^H] flunitrazepam binding parameters of cerebral cortical tissue of 3xTgAD mice.

		non-Tg	TgSED	TgEXE
Males	Bmax (pmol/mg prot)	1.77 ± 0.32	1.85 ± 0.36	1.58 ± 0.43
	Kd (nM)	3.85 ± 0.73	6.46 ± 0.69*	3.64 ± 0.69^#^

Females	Bmax (pmol/mg prot)	1.21 ± 0.43	1.45 ± 0.65	1.27 ± 0.30
	Kd (nM)	3.46 ± 1.61	4.03 ± 2.80	3.20 ± 1.08

Note: non-Tg, nontransgenic mice; TgSED, 3xTgAD mice not exercised; TgEXE, 3xTg-AD mice submitted to a daily treadmill running for five weeks. Exercise protected against the decrease of the affinity of [^3^H] flunitrazepam for its binding site in the GABA-A receptor, as showed by a low Kd. Results are the mean ± SEM, *n* = 3. Statistics: *P < .05 compared to non-Tg; ^#^ *P* < .01 compared to TgSED.
